# “It is a process” – a qualitative evaluation of provider acceptability of HIV assisted partner services in western Kenya: experiences, challenges, and facilitators

**DOI:** 10.1186/s12913-022-08024-z

**Published:** 2022-05-07

**Authors:** Wenjia Liu, Beatrice M. Wamuti, Mercy Owuor, Harison Lagat, Edward Kariithi, Christopher Obong’o, Mary Mugambi, Monisha Sharma, Rose Bosire, Sarah Masyuko, David A. Katz, Carey Farquhar, Bryan J. Weiner

**Affiliations:** 1 School of Nursing, University of Washington, Seattle, USA; 2 Department of Global Health, University of Washington, Seattle, USA; 3 PATH-Kenya, Kisumu, Kenya; 4 National AIDS and STI Control Programme, Kenya Ministry of Health, Nairobi, Kenya; 5 Kenya Medical Research Institute, Nairobi, Kenya; 6 Department of Medicine, University of Washington, Seattle, USA; 7 Department of Epidemiology, University of Washington, Seattle, USA

**Keywords:** Assisted partner services, Acceptability, HIV testing, Sexual partners, Western Kenya, Qualitative research

## Abstract

**Background:**

Assisted partner service (APS) is effective for increasing HIV testing services (HTS) uptake among sexual partners of people diagnosed with HIV with rare social harm. The acceptability of APS to HTS providers is important for the quality and effectiveness of APS delivery. Within a larger ongoing implementation science study of APS in western Kenya, we qualitatively evaluated the provider acceptability of APS.

**Methods:**

From May–June 2020, we conducted virtual, semi-structured in-depth interviews with 14 HTS providers recruited from 8 of 31 study health facilities in Homa Bay and Kisumu counties. Participants were selected using criteria-based purposive sampling to maximize variation on patient volume (assessed by the number of index clients tested for HIV) and APS performance (assessed by sexual partners elicitation and enrollment). Interviews inquired providers’ experiences providing APS including challenges and facilitators and the impact of contextual factors. Data were analyzed using an inductive approach.

**Results:**

Overall, HTS providers found APS acceptable. It was consistently reported that doing APS was a continuous process rather than a one-day job, which required building rapport and persistent efforts. Benefits of APS including efficiency in HIV case finding, expanded testing coverage in men, and increased HIV status awareness and linkage to care motivated the providers. Provider referral was perceived advantageous in terms of independent contact with partners on behalf of index clients and efficiency in partner tracing. Challenges of providing APS included protecting clients’ confidentiality, difficulty obtaining partners’ accurate contact information, logistic barriers of tracing, and clients’ refusal due to fear of being judged for multiple sexual partners, fear of breach of confidentiality, and HIV stigma. Building rapport with clients, communicating with patience and nonjudgmental attitude and assuring confidentiality were examples of facilitators. Working in rural areas and bigger facilities, training, supportive supervision, and community awareness of APS promoted APS delivery while low salaries, lack of equipment, and high workload undermined it.

**Conclusions:**

HTS providers found APS acceptable. Delivering APS as a process was the key to success. Future scale-up of APS could consider encouraging provider referral instead of the other APS methods to improve efficiency and reduce potential harm to clients.

## Background

In Kenya, approximately 1.3 million adults were living with HIV in 2018 [1]. However, only 79.5% knew their HIV-positive status, which lagged behind the first “95” of the UNAIDS 95–95-95 goal [2]. A recent randomized controlled trial in Kenya reported HIV positivity of 35% among sexual partners of HIV-positive individuals [3], much higher than the HIV prevalence in the general population in Kenya (4.9%) [1]. And 13% of these sexual partners had never had HIV testing [3]. Studies have shown the effectiveness of assisted partner service (APS) in increasing HIV testing and linkage to care [3–7]. Recently, WHO issued guidelines with a strong recommendation of offering APS to all people with HIV [8]. Currently, APS has been rolled out as standard of care in Kenya.

APS is a voluntary process where the sexual partners of consenting HIV-positive index clients are informed of their potential exposure to HIV transmission with the assistance of a trained provider. APS takes three forms: *provider referral*—providers confidentially contact partners and inform them of potential HIV exposure with the index client’s consent without revealing the identity of the index client; *contract referral*—index clients agree to inform their partners within a time frame and if they are not able to do so, provider would inform their partners; and *dual referral*—providers and index clients contact and inform the partners together [9].

Acceptability is one of the important components in feasibility studies and key considerations in program design and evaluation in implementation science [10, 11]. While effectiveness studies examine the effects of an evidence-based intervention in the real world, feasibility studies demonstrate the experience and perceptions of the stakeholders involved in the implementation, revealing contextual factors that facilitate or impede the intervention to be implemented or scaled up and indicating strategies to optimize the intervention. According to Bowen et al., acceptability looks at how the service recipients and deliverers react to the intervention, or to what extent they are satisfied with receiving or delivering this service [10]. Acceptability can be assessed quantitatively using questionnaires or scales, or qualitatively with individual interviews or focus groups. It can be evaluated before, during or after the intervention to look at the participants’ anticipated or experienced responses to the intervention [11].

Two qualitative studies conducted in Kenya have revealed barriers and facilitators for implementing APS such as clients’ fear of relationship ending, stigma, and trust between clients and HIV counselors [12, 13]. However, neither examined acceptability of APS to the service providers and challenges of delivering APS from the providers’ standpoint. As part of a larger ongoing implementation science study [14] which aimed to determine the effectiveness, feasibility, implementation fidelity, integration, and cost of APS when integrated in existing HIV service delivery in western Kenya, we qualitatively evaluated the acceptability of APS from the perspective of HIV testing services (HTS) providers. We explored their experience of providing APS, the barriers and the facilitators, and how contextual factors influenced their experience.

## Methods

### Study sites and participants

This study was nested within the APS scale-up study, a larger ongoing implementation science study (R01AI134130) [14] that aimed to scale-up APS in Kisumu and Homa Bay, two counties with the highest HIV prevalence (17.5%, 19.6%, separately) in Kenya [1]. In this study, HTS providers ask women who tested HIV positive to identify their male sexual partners, provide contact information of partners, and choose one of the three referral methods (contract referral, provider referral and dual referral) after providing adequate information on all the choices. The contacted male partners were further invited to receive APS for their other female sexual partners. Offering clients free choice of APS allowed us to see a real-life picture of preference for APS methods. As at 31st March 2021, a total of 2,538 female index clients who were tested HIV positive had been enrolled and named 8,487 male sexual partners. Of the elicited male partners, 7,585 had been successfully traced and enrolled in the study, and 3,079 were found to be HIV positive. These HIV-positive male partners further elicited 7,746 female sexual partners with 5,544 being non-index. Among those who received APS, over 80% of the female index clients and male partners who elicited partners chose provider referral. Therefore, our interviews had a particular focus on participants’ acceptance of provider referral compared to the other APS methods.

HTS providers are lay workers trained and certified by the Ministry of Health to provide HIV Testing Services at the health facilities and community, using provider-initiated testing and counselling, voluntary counselling and testing, targeted family and partner testing and other novel HTS approaches. The providers must be certified by National AIDS and STIs Control Programme (NASCOP) and have a diploma in social science or counseling psychology to provide HTS in Kenya. They were either employed by the CDC and USAID implementing partners in collaboration with the Ministry of Health in Kenya or employed directly by APS Study to ensure study procedures were appropriately delivered and data were timely collected and uploaded. All the programs train their providers on HTS using the latest National HTS guidelines and on APS documentation and reporting.

To examine the acceptability of implementing APS among HTS provides, we purposefully selected 8 of the 31 health facilities to maximize variation on two criteria: APS service performance and patient volume. These selection criteria were used to facilitate exploration of whether HTS providers’ experience of APS differed between high- and low-performing or high- and low-volume facilities. APS performance was measured by the partner elicitation ratio (the number of male partners identified per female index client) weighted by the percentage of identified male partners enrolled in the study, and facility volume was measured by the number of female clients tested for HIV in the facility. A high weighted partner elicitation ratio signified high performance, and a high number of tested female clients signified high volume. Calculations were based on the cumulative data from January to December 2019. Four facilities (one high-performance high-volume, one high-performance low-volume, one low-performance high-volume, one low-performance low-volume) from each county were included. At each facility, based on the facility staffing, one to four HTS providers who had received APS trainings, worked on APS for at least 3 months and were willing to participate in the study were included in the interviews.

### Study procedures

Eligible HTS providers were invited to participate in the semi-structured individual in-depth interviews (IDIs) conducted between May and June 2020. Interested participants were asked to sign the consent form and schedule the interview using a virtual platform. An experienced qualitative researcher (MO), who was independent of the implementation team, conducted the interviews in Kiswahili, Luo or English, based on participants’ language preference. Participants were asked to talk in a quiet place and consent to be audio recorded. Sociodemographic characteristics including age, gender, place of residence, educational level, and length of time working as on HTS and on APS were collected. Each interview lasted one to two hours. While most were completed in a single session, several interviews required multiple sessions due to poor network, interruptions, difficult time scheduling, or participant fatigue. Recorded interviews were transcribed verbatim and translated into English. Transcripts were peer-checked by another researcher (WL) to ensure the accuracy.

### Data management and analysis

Recorded interviews and corresponding transcripts were assigned an ID numbers with identifying information removed. Data were analyzed thematically using inductive coding. After reading the transcripts carefully, one researcher (WL) developed the codebook, and another researcher (MO) revised it. After the codebook was tested with two transcripts, the two researchers coded all the transcripts independently and reached consensus through discussion. Themes that derived from research questions and newly emerged from the data were identified and discussed. Comparison analysis was further conducted to examine differences in themes and subthemes between different types of facilities. ATLAS.ti version 8.4.4 (ATLAS.ti Scientific Software Development GmbH, Berlin, Germany) and Excel were used for analysis.

## Results

### Participant characteristics

Fourteen HTS providers participated in the IDIs (Table 1). Most participants were female, over half were from rural, public facilities, and 72% were from high-volume facilities that had higher staffing. All the participants had completed postsecondary education, had worked as HTS providers for at least 16 months, and worked on APS for ≥ 8 months.

**Table 1:** Participants Characteristics (*n* = 14)

Variable	Mean (range) or n (%)
Age (years)	35 (25–52)
Sex	
Male	4 (29%)
Female	10 (71%)
Level of education	
College diploma	12 (86%)
Postgraduate	1 (7%)
Certificate	1 (7%)
County	
Kisumu	7 (50%)
Homa Bay	7 (50%)
Urban/rural facility	
Urban	5 (36%)
Rural	9 (64%)
Public/faith-based facility	
Public	9 (64%)
Faith-based	5 (36%)
Facility volume & performance	
High-volume high-performance	6 (43%)
High-volume low-performance	4 (29%)
Low-volume high-performance	2 (14%)
Low-volume low-performance	2 (14%)
Length of working time as an HTS provider (years)	5 (1.3–12)
Length of working time doing APS (years)	1.7 (0.7–3)

The main themes and subthemes revealed by the analysis concerned: 1) overall experience of delivering APS; 2) challenges and facilitators of provider referral; and 3) contextual factors affecting provider acceptability of APS.

#### Theme 1: overall experience of delivering APS

Overall, HTS providers perceived APS and provider referral as acceptable. Three providers perceived their APS experience as “good”, two reported it was not that easy, and eight stated it was both good and bad. As to provider referral, nine providers perceived it as good, and five perceived it as sometimes challenging.

##### Doing APS is a process

Although not asked, participants consistently stated that doing APS is a continuous process, not a one-day job. This is demonstrated in each step of APS, including partner elicitation, tracing, and notification. As one participant described:

*“When you are introducing APS, you don’t expect it to work on the same day you have introduced it and succeed. It will be something gradual.” (KII 018, from a high-volume high-performance public facility in rural area)*

First, clients need time to accept their HIV status, and become open to talk about sexual partners. Second, providers need to continuously follow up the index clients to elicit more because their sexual partners might keep changing. Third, tracing partners is a tortuous process with many challenges and obstacles, and once locating the partners, opening a dialogue about APS and persuading the partners to accept HIV testing also take time.

*“You know for some clients—this APS is something new to them, so some are shocked when you tell them that you want their sexual partners. So I have to create a rapport and explain to them what APS is. ... There are some who decline ... [for those] I give them time but I contact them from time to time to see if they are now ready." (KII 030, from a high-volume high-performance public facility in rural area)*

##### What the providers like about APS

One commonly reported benefit of APS was its efficiency in HIV case finding and linkage to care. With specific targets, it takes less time and resources compared to the past door-to-door testing approach.

*“The good side is that at least it is specific; I mean you just go to that person—it even saves on the resources. Maybe someone stays in somewhere and so you will just go to one person, not like you will go around the places testing from door to door.” (KII 028, from a high-volume low-performance public facility in urban area)*

In addition, by doing APS, providers were able to reach the unreached clients who do not normally access health facilities, especially males.

*“APS is concerned with these male sexual partners who are not able to be tested. … I can say on my side most of the positive clients I have achieved are from APS, especially male.” (KII 019, from a low-volume high-performance public facility in rural area)*

Having clients who are potentially HIV-exposed learn their status, receive treatment early and become virally suppressed if HIV-positive, and as a result, to benefit the whole families and curb HIV transmission in the society, has become a powerful motivation for most providers to do APS.

*“I do it to help and to leave other families happier or healthy, compared to when I know something and I just leave or I just keep calm yet within myself I know that this person could be having HIV so when I go, whether I get the person positive or negative I still find it good, I find I am happier and in my feel I have assisted.” (KII 025, from a high-volume high-performance mission hospital in rural area)*

Almost every participant mentioned they had obtained knowledge, experience and skills over time. They gained a deeper understanding of APS, learned how to approach different clients and deal with various difficult situations, and also gained higher self-efficacy to implement APS.

*“When we started we found it difficult when we go for tracing, we didn’t even have the words to tell our clients for them to accept easily and get tested. But now after being taken for trainings and support supervision, we have learnt from other side we have shared our experiences and we are finding it easier compared to when we started.” (KII 025)*

##### What the providers like about provider referral

Apart from the above-mentioned benefits of general APS, provider referral has several advantages relative to other modalities of APS. In provider referral, HTS providers are authorized to contact the partners on behalf of the index clients, leveraging their professional knowledge and communication skills. This relieves index clients’ burden of disclosing HIV status to their partners and persuading them to receive HIV testing. It protects the index clients’ confidentiality and avoids jeopardizing the index clients to intimate partner violence (IPV) or broken relationships.

*“if you want to tell me about contract referral, there is that fear, somebody will tell you, ‘if I go and do it alone, how will I deliver it? How will I start telling my partner?’ … So you find provider is worth doing because with the skills that we have and the guidelines that we have been given or that they have set for us makes it very easy for us now to reach them as opposed to the other methods.” (KII 023, from a high-volume high-performance mission hospital in rural area)*

Moreover, tracing and informing the partners independently without relying on the index clients’ engagement as in dual or contract referral makes it more efficient. It helps the providers identify more HIV cases within the same time period, reach the target easier and gain job satisfaction.

*“In contract referral as the names suggest, you have to sign something with the [index] client that you are going to do it within a particular period and... because our catchment area is big, some of these clients are maybe out of our catchment area. So getting the client might take long. ...So that makes me like the provider referral rather than contract.” (KII 024, from a high-volume high-performance mission hospital in rural area)*

Although it is the clients who determine which referral method works best for them, nearly half of the providers preferred provider referral because of these advantages. Several participants believed all methods should be used to serve different situations.

#### Theme 2: challenges and facilitators of provider referral

In provider referral, it is crucial to assure the confidentiality of both index clients and sexual partners. Clients’ curiosity and demands for more information often put HTS providers in a dilemma. Some partners wanted to know where the HTS provider got their phone numbers, while some index clients wondered about the HIV status of partners identified. Providers shared their strategies to deal with this dilemma:

*“I might say we have a suggestion box and your number was picked from the suggestion box. Or say maybe ‘I’m calling from maybe the Ministry of Health, we had a directory so this number we have picked from a directory or Safaricom subscribers.’” (KII 024)*

*“The index demands the results sometimes and this has been a challenge on my side. …I will tell the client ‘Since you know this guy, and you have the number, just call him and he will tell you our experience and the outcome.” (KII 022, from a high-volume high-performance mission hospital in rural area)*

##### Challenges and facilitators of partner elicitation

Half of participants found eliciting sexual partners from the index clients not easy, because “it is a sensitive part of a human being” (KII 023), and “telling someone strange about your sexual partner is not a joke; it is a serious thing” (KII 022). Most clients were not able to open up when they just knew that they were HIV-positive. Some feared of a breach of confidentiality; others worried that they might be judged for having multiple sexual partners.

*“The moment you try to elicit more sexual partners, they feel that you will look at them like they are not morally upright, so there is that fear of judging them.” (KII 016, from a high-volume low-performance public facility in urban area)*

To address these challenges, several participants emphasized the importance of building good rapport with the clients, communicating with them in a nonjudgmental attitude, and assuring them of the confidentiality.

*“As a counsellor, you will need to do a proper counseling to make the [index] client understand that it is ok to have even up to 10 sexual partners... So the moment you put that partner at peace and if you develop that good relationship, then with time you will get more.” (KII 016)*

Providers’ patience and continuous follow-up with clients were of equal importance. “You just give them time” (KII 027), and as your relationship became close, they would be able to open up. Another tactic was to collect the information before clients obtained their HIV testing result, because once they knew they were HIV-positive, they would fear disclosing their status and thus only provide the contact of their spouse. It was also helpful to obtain assistance from colleagues, community health volunteers, or peer educators who were more familiar to clients.

##### Challenges and facilitators of partner tracing, informing and providing HIV testing

According to the partner tracing standard operating procedure (SOP), HTS providers are supposed to contact the partners by phone first up to three times, then conduct a physical tracing. If that still fails, two additional tracing attempts either by phone or in person should be made.

In the interviews, six participants reported that their experience of tracing or informing the partners of their potential exposure to HIV was “not easy” or “hard,” five said their experience was “both easy and hard,” and two said it was “easy” or “not difficult.”

Multiple participants experienced obstacles attributable to incorrect information provided by the index clients, in terms of phone number, locating information, or the character of the partner.

*“What can prevent me from doing provider referral is when the index client gives me the contacts of these sexual partners, most of these contacts are not going through; or the location she gave me is not the true location.” (KII 018)*

In this case, building a good relationship with the index client so that she/he can provide the real picture is critical. However, sometimes even the index clients did not have the contact information of some partners (e.g., those casual ones a long time ago).

Poor weather, distance, geographical barriers and unavailable transport also created barriers for physical tracing.

*“You know where I work it rains a lot. And sometimes you don’t have gumboots and you need to go look for a client where it is so muddy…” (KII 020)*

*“You find that it is also hard in that during the physical tracing, you need to go out and the sun is so scorching and you have to walk very far where the motorbikes cannot reach.” (KII 021, from a low-volume high-performance mission hospital in urban area)*

Challenges also came from the sexual partners. Sometimes partners were busy with work and hard to schedule with; sometimes partners had moved to a new place but the index clients did not know. Providers also suffered from the partners’ suspicion about their intention to call them, or the partners’ defensive behaviors when they felt offended by a stranger trying to learn about their sexual life.

*“Some will ask you ‘Where did you get my number?’ And they will insist and become very rude. Some throw nasty words at you or they will insult you: ‘You are a con woman. You want to con me!’” (KII 027, from a high-volume low-performance public facility in urban area)*

*“If you can involve in contacting these male sexual partners, sometimes they feel 'Why are you asking me my sexual partners?’ Sometimes they think that you want to be involved with that female sexual partner in sex activity.” (KII 019)*

And many setbacks were from the partners’ refusal and hostility due to HIV-related stigma. Some sexual partners did not pick up the phone when they saw the number was from a HIV-related facility or even put the number into blacklist. If the HTS provider visited them in person, some partners hid, and some chased them away.

*“I have been working in this community, they know that this is the person show tests for HIV at the facility. So when they see me, they just run away. They don’t want me to go to their home.” (KII 021)*

A couple of providers expressed safety concerns of working in the field because they might be threatened, attacked, mistreated, or sexually harassed, even though cases were rare.

*“In some areas there is a lot of insecurity. You never know whether you will be attacked or not. … Like a case I tried to follow and the partner came out with a panga. And I ran away.” (KII 016)*

According to participants, around 50–98% of the partners would agree to test for HIV once being informed of their potential HIV exposure. Some partners refused because they were not ready for HIV testing; some already knew their status. The key to success was to create good rapport, help them understand the importance of knowing HIV status and also provide encouragement and support.

*“It is how you create the rapport with the client. If you are harsh, rude, the client will not agree but if you talk to the client slowly and tell them the benefits of knowing their status, you know, some of them will just agree.” (KII 020)*

To address the partners’ refusal to HIV testing services, some participants used the strategy of starting the conversation outside HIV to build rapport and bring up HIV later:

*“Before you do anything more with the client, you have to ask for the clients’ health first. So after contacting the client, promote other health services so that when you talk also about HIV, they will not reject. That is the method I have been using.” (KII 019)*

Others pretended to do routine door-to-door testing with the target partner in mind to avoid disclosing the clients’ identity. Sometimes changing the gender or age of the HTS providers also worked.

*“I link a fellow provider who is a female to deal with this male partner who is difficult. … Yes, men are more open to females.” (KII 022)*

*“Maybe a client will see me as a bit elderly and when we have a younger person to talk to the youth, they will accept because they will feel like this provider is the same age as them.” (KII 023)*

Another challenge was cost. Over half of the providers felt the cost of tracing the partners was high. Although they were provided with airtime for reimbursement for transport, the actual cost was often beyond the budget, where they had to do it out of pocket or suspend the service until they were refilled.

*“When I am doing it out of my pocket then I might find it sometimes difficult and I feel like 'ah, today I might not do this because I don’t have enough cash.' So I will weigh, between myself, my family and the client...” (KII 018)*

Therefore, several participants suggested the facility ensure adequate airtime and provide fund for transport beforehand or reimburse it sooner. Additionally, considering that doing APS is a time-consuming process and every client is unique, nearly half of the participants claimed that the SOP needs to be improved to allow flexibility with different kinds of clients and situations.

*“It (the protocol) shouldn’t be too limited because sometimes you will find that you have filled the tracing form and the client is still yet not cooperative. And remember you should do it 6 times and then give up on this client. But again you think this client needs help… so I think they should not be so strict.” (KII 017, from a low-volume low-performance public facility in rural area)*

#### Theme 3: contextual factors affecting provider acceptability of APS

##### Rural versus urban residence

Ten participants mentioned tracing partners for APS was easier in rural areas because their residences in rural areas tends to be more permanent compared to people in urban areas who have to relocate frequently due to job or rental unit changes.

*“In rural areas, homes where the clients come from never change. And most people don’t go to work … compared to towns somebody might be living in Migosi today, next time you go you find that she moved to somewhere where neighbors do not even know. Another thing, when you are going to test this client mostly during the day, people in town they go to work and that is the time when we are also at work, so scheduling with this person is hard.” (KII 025)*

One participant said that due to concerns of privacy some people prefer going to facilities that are farther or more interior for HIV related services, which also made doing APS in rural area easier:

*“You will find someone starting to go to a facility that is far much interior… Most of the clients don’t want to go to facilities that are along the road… So doing APS in a facility that is interior like a dispensary is easier than doing APS in facilities that are situated along the road.” (KII 018)*

By contrast, one participant thought it was harder working in rural areas because of people' priority on basic life necessities and relatively low level of education and health literacy:

*"In rural, you will find people who are not literate. Giving information is like you are digging your own grave [laugh]. ... like now where I work the level of poverty is high. Will someone allow you to give them information on HIV and yet they are hungry? … Some will tell you, 'instead of telling me this, give me money so that I can feed first.'” (KII 023)*

##### Facility volume and number of providers

When asked about how facility and number of providers affected APS, six participants responded that APS worked better in bigger facilities where the patient flow and the number of providers are larger, and the range of service and drug inventory is broader.

*"In small facilities, the flow of clients is not there, the rate of positivity is also down. But in a bigger hospital like the county hospital or the sub county hospital, the number of clients who normally come for the test is high and so getting a client from this large number of clients is not a problem." (KII 018)*

Six participants believed that the size of the facility does not affect APS performance and what really matters is the staffing, quality of service, facility management and teamwork*.* Eight participants pointed out the significance of manpower: When there are too few HTS providers in a high-volume facility, the workload and quality of service would become a problem.

##### Salary, incentives and working conditions

Over half of participants thought their salaries or incentives need to be increased. Three participants working in rural facilities mentioned they had to work during holidays or go without leave days.

*“The remuneration is a bit low and it is not very easy to make ends meet for our families. This is a discouragement.” (KII 016)*

Participants also expressed the need for raincoats, gumboots, umbrellas, blood pressure machines and power backup in physical tracing.

##### Trainings, support supervision

Participants consistently perceived the trainings and support supervision they had received helpful. During the trainings, they were able to exchange experiences, share problems, figure out solutions and make strategic plans together, which “smoothened their work.” The supervisors were also supportive. They helped pinpoint any ineffective actions that the providers were not aware of before, remind them what other actions need to be done, and assist with handling tough clients or situations. However, 9 out of the 14 participants pointed out the trainings were not adequate and suggested adding more refresher trainings.

##### Community awareness of APS

The level of community awareness of APS also affected HTS providers’ acceptance of implementing APS. All but three participants said most people in the community had heard about APS, mainly from HTS providers, health talks offered at the facility, or outreach services.

*“We normally give health talks. We normally have some outreaches. We have some satellites and so when we go there we talk about the APS. Within the facility every Thursday once a month a counsellor talks about APS. So the community they understand it.” (KII 022)*

Six providers said most people reacted positively when they were introduced to APS, or they refused at the beginning but gradually accepted it. Four providers experienced mixed reactions from the community: While some say APS is good in that it helps people know their status and linked to care, others do not accept it due to fear of breach of confidentiality, concerns of broken relationship or fear of being judged for having multiple sexual partners. The others encountered mostly negative reactions from clients.

## Discussion

Contrary to most prior APS related qualitative studies that focused on clients’ acceptance and perceptions of APS, our study revealed HTS providers’ unique perspective on APS acceptability. Through the interviews, we elicited their particular experience of delivering APS to HIV-positive clients, the challenges and obstacles in service delivery, and their strategies and suggestions to facilitate APS delivery. The relative advantages of provider referral compared to other APS methods were also identified.

Overall, the HTS providers found APS acceptable. The altruistic benefits of APS were strong contributors to this acceptability. Helping clients know their HIV status, linking them to care, improving the wellness of the whole family and reducing HIV transmission in the population were mentioned by many HTS providers as their motivation to provide APS. This altruism was also reported in Quinn et al.’s study [15]. Another factor that contributed to HTS providers’ acceptability was the efficiency of APS in HIV case finding and linkage to care. By precisely targeting the sexual partners of HIV-positive index clients, HTS providers were able to identify more HIV-positive individuals and link them to treatment faster. They were also able to reach and involve more male clients to HIV testing and care who seldomly visited health facilities.

Our participants described many challenges when delivering APS. Although the barriers due to cost, weather, distance, geographic obstacles, and transport were also evident, the most prominent challenges were from the clients. For instance, as similarly reported in other studies [9, 15–17], it was not easy to obtain accurate contact information of the sexual partners from the index clients, and tracing sexual partners was often challenging due to their relocation. Additionally, many setbacks were from the index clients’ hesitancy to open up about their sexual partners and the partners’ refusal and hostility when contacted due to distrust and HIV-related stigma. These barriers reflected the clients’ fear of breach of confidentiality and disclosure of HIV status. Many previous studies had reported clients’ concern of confidentiality and the fear of stigma and discrimination, fear of blame and violence, and fear of broken relationship if their HIV-positive status was disclosed [12, 13, 17, 18]. Therefore, it is critical for HTS providers to assure clients’ confidentiality when delivering APS. In our study, HTS providers shared strategies such as telling the sexual partners they got their numbers from a directory of certain customers, and pretending they were doing a door-to-door testing to protect the sexual partners’ identify. These strategies could be adapted by other HTS providers to improve APS acceptance.

To overcome all these difficulties and achieve successful APS delivery, our participants emphasized the importance of building good rapport with clients, communicating with clients with patience and nonjudgmental attitude, and following up with clients with perseverance. As one of our subthemes indicated, “doing APS is a process.” APS could not be achieved in one day, and rushing the process would undermine successful HTS delivery. This is an important implication and should be noted by future APS implementation and scale-up programs.

Another major finding of our study is the high acceptability of providers referral compared to other referral methods among HTS providers. This preference was ascribed to its relative advantages including efficiency in contacting the sexual partners, relieving the index clients from the burden of disclosing HIV status, leveraging providers’ professional knowledge and communication skills, and reducing the risk of IPV. In another qualitative study examining acceptance of partner notification for HIV positive clients in Ethiopia [18], HIV counselors also favored provider-assisted partner notification rather than client notification, because health professionals can provide better information and counseling and prevent misunderstanding and conflicts that may be induced by client notification. In a recent cross-sectional survey by Samson et al. among Kenyan HIV infected clients [19], a high proportion of the clients chose provider referral as the preferred referral method, which echoed the advantages of provider referral in professionalism and protection of clients’ confidentiality. Empirical research has demonstrated that provider referral was the most effective approach in delivering information to partners and linking them to testing and care [16, 20, 21]. Although some studies reported passive referral was clients’ most preferred referral method [19–22], lack of communication skills and fear of broken relationships often prevent them from informing partners by themselves [20]. Therefore, leveraging the professional knowledge and communication skills in provider referral would improve acceptability of APS among service providers and protect clients from potential harm. But one caveat is that when the index client only has one sexual partner, provider referral will inevitably disclose the index client’s identity [12, 15].

As to contextual factors, we found that funding, human and material resources, training, and supervision are crucial for successful APS delivery and substantially affect HTS providers’ acceptance of APS. While effective trainings and support supervision facilitated APS delivery, delayed reimbursement of transport and airtime, low salaries, lack of equipment, and high workload undermined it. Previous studies have reported lack of training, resources, or funding as structural barriers of APS delivery and scale-up [15–17, 22, 23]. For APS to be applied in broader settings, it is important to ensure adequate funding and training for the service deliverers. We also observed increased community awareness of APS. A study conducted in Kenya in 2016 had reported that lack of community awareness impeded the uptake of APS [12], when APS was still a novel concept. Currently, more people have been aware of APS and willing to accept it, which also increased HTS providers’ acceptance of APS.

### Strengths and limitations

This study had several strengths. First, it revealed the acceptability of APS from the particular perspective of the service deliverers, which created important implications for the improvement of quality and efficiency of APS delivery. Second, the criteria-based purposive sampling used in this study maximized the sample’s representativeness of different types of facilities. Third, one coder from the local community who also conducted the interviews ensured the interpretation was not divorced from the interview context and the local culture. And the frequent peer debriefing and dialogue between the coders ensured credibility. We also had limitations. First, the COVID-19 epidemic has restricted in-person contact and thus all interviews were conducted virtually. This has led to missing information including background environment and nonverbal signals. However, audio-recording the interviews and verbatim transcription preserved the essential elements of the conversations and provided valuable information about APS delivery. Second, the sample size was small and restricted our ability to analyze the similarities and variances between different types of facilities. Future research can consider expanding the sample size from each type of facility to increase the power of comparison analysis.

## Conclusions

The HTS providers in our study perceived APS especially provider referral acceptable. Future scale-up of APS should take into consideration the identified barriers and facilitators that affected providers’ acceptance to improve APS delivery. Patience, persistence, and good rapport with clients should be encouraged, and the importance of assuring clients’ confidentiality should be emphasized. Ensuring adequate funding, training and resources can improve providers’ motivation to deliver APS. Promoting provider referral instead of other referral methods may increase the efficiency and safety of partner notification, HIV-positive case finding and linkage to care.

## Data Availability

The datasets (transcripts) used and analyzed during the current study are available from the corresponding author on reasonable request.

## Abbreviations

APS: Assisted partner services
ART: Antiretroviral therapy
HTS: HIV testing services
IDI: In-depth interviews
IPV: Intimate partner violence
SOP: Standard operating procedure
